# The quantitative proteomic response of *Synechocystis* sp. PCC6803 to phosphate acclimation

**DOI:** 10.1186/2046-9063-9-5

**Published:** 2013-02-26

**Authors:** Matthew A Fuszard, Saw Yen Ow, Chee Sian Gan, Josseilin Noirel, Nigel G Ternan, Geoff McMullan, Catherine A Biggs, Kenneth F Reardon, Phillip C Wright

**Affiliations:** 1 BSRC Mass Spectrometry and Proteomics Facility, Department of Chemistry, University of St Andrews, St Andrews, KY16 9ST, UK; 2 ChELSI Institute, Department of Chemical and Biological Engineering, University of Sheffield, Sheffield, S1 3JD, UK; 3 Agilent Technologies, Singapore, Singapore; 4 School of Biomedical Sciences, University of Ulster, Coleraine, County Londonderry, BT52 1SA, UK; 5 Department of Chemical and Biological Engineering, Colorado State University, Fort Collins, CO, 80523-1370, USA

**Keywords:** Cyanobacteria, iTRAQ, Phosphate acclimation, Phycobilisome, Pentose phosphate pathway, Ribose sugars, *Synechocystis*

## Abstract

**Background:**

Inorganic phosphate (P_i_) is a critical nutrient for all life and is periodically limiting in marine and freshwater provinces, yet little is understood how organisms acclimate to fluctuations in P_i_ within their environment. To investigate whole cell adaptation, we grew *Synechocystis* sp. PCC6803, a model freshwater cyanobacterium, in 3%, and 0.3% inorganic phosphate (P_i_) media. The cells were allowed to acclimate over 60 days, and cells were harvested for quantitative high throughput mass spectrometry-based proteomics using the iTRAQ™ labelling technology.

**Results:**

In total, 120 proteins were identified, and 52 proteins were considered differentially abundant compared to the control. Alkaline phosphatase (APase) activities correlated significantly (p < 0.05) with observed relative PhoA abundances. PstS1 and PstS2 were both observed, yet PstS1 was not differentially more abundant than the control. Phycobilisome protein abundances appeared to be coordinated, and are significantly less abundant in 0.3% P_i_ than 3% P_i_ cultures. Also, the central metabolic cell function appears to have shifted towards the production of (NADPH) reducing energy and nucleotide sugars.

**Conclusions:**

This acclimation response bears strong similarity to the previously reported response to nitrogen deprivation within *Synechocystis* sp. PCC 6803. However, it also demonstrates some characteristics of desiccation stress, such as the regulation of fatty acids and increased abundance of rehydrin in the 3% P_i_ culture.

## Background

Cyanobacteria comprise one of the largest groups of prokaryotes. They are found in a wide variety of niches, including terrestrial and hypersaline environments, and in oceans, lakes and rivers. *Synechocystis* sp. PCC 6803 (hereafter *Synechocystis*), a model freshwater cyanobacterium, has strong potential biotechnology applications in areas as diverse as biofuels [1], biopolymers [2] and other secondary metabolites [3]. Due to this biotechnological interest, its general applicability as a model organism for CO_2_ fixation and environmental stress responses has been the subject of post-genomic investigations [4-8], including proteomics investigations using both 2-DE [8] and shotgun proteomics workflows [7].

For most microorganisms, the preferred source of the essential nutrient phosphorus is inorganic phosphate (P_i_). However, phosphate is the planet’s least abundant naturally occurring mineral [9]. P_i_ limitation occurs for sustained periods in many geographically dispersed freshwater lakes and marine sites. To date, however, there have been relatively few investigations into the cell-wide response of microorganisms to this form of nutrient stress, particularly over longer periods that reflect adaptation rather than short-term response. An understanding of these processes will be of use not only in the basic understanding of microbes and the niches they occupy, but could also be applied to develop biomarkers to follow stages in the phosphorus biogeochemical cycle within aquatic environments. In addition, the identification of novel proteins associated with P_i_ uptake could be exploited in the removal of P_i_ for water quality improvement, whilst enzymes involved in the catabolism of organophosphorus molecules are of interest in the pharmaceutical and agribiotech industrial sectors.

There has been a range of phosphate starvation and limitation studies in bacteria carried out through cDNA microarrays to investigate global transcriptome changes [10-13] and qualitative overview analysis using 2-DE workflows to study broad proteome changes [14-17]. To date, there have only been two high-throughput quantitative proteomic assays of P_i_ limitation in cyanobacteria, focusing on the marine species *Prochlorococcus*[18,19]. While it is tempting to compare the observations obtained from these studies, comparison among datasets has also been further complicated by the different types of experiments conducted, which range from evaluation of the short-term responses to starvation conditions (shift to P-free medium) to studies of adaptation to P_i_ limitation. However, one of the most common observations in all of these studies is the up-regulation of *pho* regulon genes, as seen in studies conducted on *Bacillus subtilis*[15], *Escherichia coli*[19], *Prochlorococcus* spp. [13,18,19], *Synechococcus* WH8102 [20] and *Corynebacterium glutamicum*[21].

Unlike other prokaryotic systems, reports on P_i_ limitation responses in cyanobacteria are relatively few. When available, these studies consist more generally of short-term P_i_ shock experiments with a shift from replete to P_i_ free medium [10,13,20,22-26]. However, these studies have revealed that the phosphate-sensing system in *Synechocystis* is modulated by SphS (sll0337; histidine kinase; analogous to PhoR of *E*. *coli*) and SphR (slr0081; response regulator; analogous to PhoB of *E*. *coli*) [27], which form a two-component signal transduction system. Also, an in-depth investigation of P_i_-binding proteins within *Synechocystis* showed differential regulation, functional roles and P_i_ transport characteristics for its two ABC-type phosphate transporter proteins, PstS1 and PstS2 [22].

Inactivation of *phoU*, one of the genes encoding phosphate-specific transport system regulatory proteins, caused an increase in both inorganic phosphate (Pi) uptake and polyphosphate accumulation rates [27]. It has been shown that under phosphate starvation, the *Synechocystis pho* regulatory system responds by increasing its transcript abundance by more than 7-fold compared to normal P_i_ replete conditions [10]. It has been suggested that high light levels also triggered the *pho* regulon, since light accelerated phosphate assimilation despite limited cell capacity to perform the task [28]. Suzuki *et al*. used DNA microarrays to investigate the *Synechocystis* phosphate starvation response [10] and found that the genes encoding the ABC-type phosphate transporter (*sphX*, PstS1 and PstS2 clusters) and extracellular nuclease (*nucH*) were significantly up-regulated in addition to the alkaline phosphatase (*phoA*) gene. The gene encoding a periplasmic binding protein (*urtA*) was down-regulated. In a separate study under similar conditions, significant up-regulation of the genes for inorganic pyrophosphatase (*ppa*) and exopolyphosphatase (*ppx*) was observed [29].

The goal of this work was to study the adaptation of *Synechocystis* to phosphate limitation using a quantitative shotgun proteomics workflow [30,31]. The proteomes of *Synechocystis* grown in different phosphate concentrations (3% and 0.3% of the non-limiting level in BG-11 medium) were quantitatively measured using iTRAQ reagents. Coupled with enzyme assays, this investigation forms the basis for understanding global cellular responses, such as the central metabolism and biosynthetic pathways, of *Synechocystis* in phosphate-limited environments. This study is both novel and timely as it specifically deals with an in-depth analysis of P_i_-depletion over different levels of phosphate, and over a longer timescale, than has been explored by others. The results of this study provide insights into the ways in which cellular processes are directly influenced by ambient phosphorus concentrations.

## Results and discussion

### Growth characteristics

In phosphate-depleted conditions, the growth rate of *Synechocystis* was significantly impaired (Additional file 1: Figure S1), with noticeable reduction of colour within the flasks (data from observation, image not available). The control culture reached an OD_730_ of 7 at the end of the 60-day cultivation, whereas the phosphate-limited cultures only reached a maximum OD_730_ of 0.9 (for 3% P_i_ level) and 0.5 (for 0.3% P_i_ level). Their specific growth rates were calculated as 0.20 day^-1^ for the control culture, 0.012 day^-1^ for 3% P_i_ culture and 0.002 day^-1^ for 0.3% P_i_ culture. This impairment in growth with sub-optimal P_i_ levels was expected, and agrees with the trend observed for other organisms [21,32,33].

### Proteome overview

**Figure 1:**
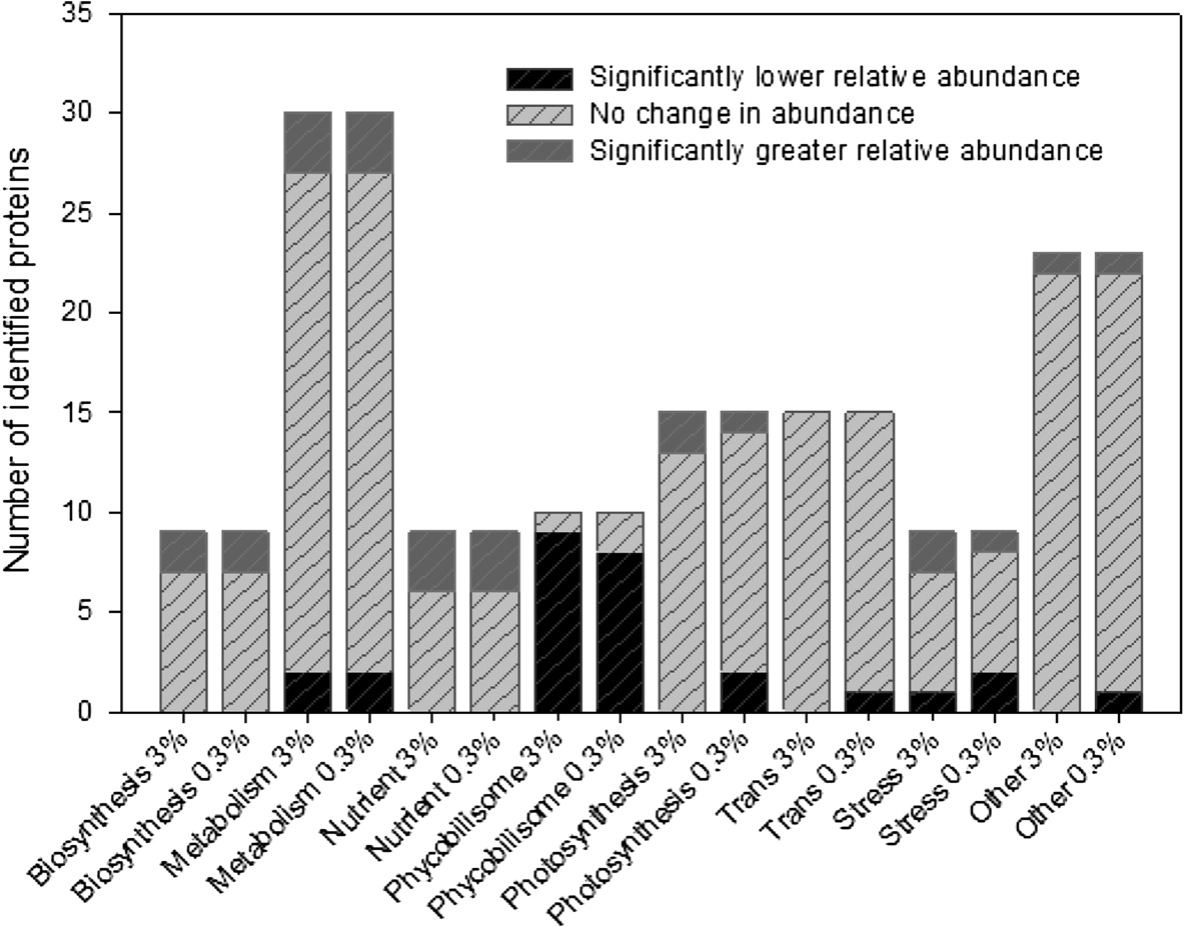
**Stacked bar chart highlighting the proportion of differentially abundant proteins found within 3% and 0.3% P**_**i **_**cultures.**

A total of 120 proteins were identified and quantified with two or more peptides (Additional file 2: Table S1). A quarter of these were associated with central metabolic functions (glycolysis, pentose phosphate pathway, glycogenesis, glyconeogenesis and carbon fixation) (Additional file 1: Figure S2). A significant fraction (19.2%) was associated with uncharacterised and hypothetical proteins, and 21% were associated with photosynthesis. In the study, 13 detected proteins were more abundant in the 3% P_i_ culture, whereas the abundances of 12 proteins were observed to have increased within the 0.3% P_i_ culture (Table 1). Conversely, we observed 13 proteins to be lowered in abundance within the 3% P_i_ culture, while 16 other identified proteins were less abundant in the 0.3% P_i_ culture (Table 1, Figure 1, Additional file 2: Table S1).

**Table 1:** Differentially abundant proteins related to control cultures

***Protein details***					***Relative abundance***			
**Functional category**	**ORF**	**Protein name**	**Gene name**	**# peptides**	**3% P**_**i**_	**SD**	**0.3% P**_**i**_	**SD**
Nutrient assimilation and regulation	sll0654	Alkaline phosphatase	*phoA*	3	2.72^b^	0.09	3.74^b^	0.28
	sll0680	Periplasmic Pi-binding protein	*pstS1*	7	1.04	0.23	2.25^b^	0.60
	slr1247	Periplasmic Pi-binding protein	*pstS2*	15	2.99^a^	0.34	4.69^a^	0.15
	slr1622	Inorganic pyrophosphatase	*ppa*	11	1.32^b^	0.05	1.38	0.02
Light harvesting and photosystems	sll1578	C-phycocyanin alpha chain	*cpcA*	169	0.67^a^	0.06	0.28^a^	0.03
	sll1577	C-phycocyanin beta chain	*cpcB*	311	0.75^a^	0.09	0.30^a^	0.02
	sll1580	Phycobilisome 32.1 kDa linker polypeptide, phycocyanin-associated, rod 1	*cpcC1*	9	0.58^b^	0.07	0.22^c^	0.06
	sll1579	Phycobilisome 32.1 kDa linker polypeptide, phycocyanin-associated, rod 2	*cpcC2*	41	0.71^a^	0.13	0.28^b^	0.03
	slr2051	Phycobilisome rod-core linker polypeptide	*cpcG*	21	0.73^b^	0.05	0.29^a^	0.04
	slr2067	Allophycocyanin alpha chain	*apcA*	85	0.56^a^	0.03	0.33^a^	0.01
	slr1986	Allophycocyanin beta chain	*apcB*	64	0.61^a^	0.01	0.42^a^	0.02
	sll0928	Allophycocyanin-B	*apcD*	9	0.55^b^	0.03	0.34^a^	0.03
	slr0335	Phycobilisome LCM core-membrane linker polypeptide	*apcE*	21	0.56^a^	0.07	0.31^a^	0.00
	sll0258	Cytochrome c-550	*psbV*	27	0.88	0.04	0.55^a^	0.04
	slr1643	Ferredoxin NADP reductase	*petH*	10	0.86	0.05	0.73^b^	0.07
	slr0729	Thylakoid-associated protein slr0729	*slr0729*	21	1.39^b^	0.12	1.28	0.04
	ssl2501	Uncharacterized thylakoid-associated protein ssl2501	*ssl2501*	7	2.97^a^	0.04	2.82^a^	0.29
Central metabolism	slr1793	Transaldolase	*tal*	27	1.28^b^	0.05	2.05^a^	0.01
	sll0329	6-phosphogluconate dehydrogenase, decarboxylating	*gnd*	13	1.49^a^	0.02	1.88^a^	0.16
	slr1843	Glucose-6-phosphate 1-dehydrogenase	*zwf*	6	1.54^b^	0.10	1.99^b^	0.10
	slr0394	Phosphoglycerate kinase	*pgk*	8	0.60^b^	0.05	0.86	0.05
	slr0009	RuBisCO large subunit	*rbcL*	46	0.64^b^	0.02	0.64^b^	0.10
	slr0012	RuBisCO small subunit	*rbcS*	24	0.82	0.03	0.38^a^	0.00
Biosynthesis	slr1994	3-oxoacyl-[acyl-carrier-protein] reductase 2	*fabG2*	5	2.17^b^	0.01	2.83^a^	0.05
	sll1852	Nucleoside diphosphate kinase	*ndk*	4	1.00	0.12	2.07^b^	0.15
	sll1356	Glycogen phosphorylase	*glgP(2)*	4	1.51^b^	0.13	2.00^c^	0.17
Transcription, translation and stress	sll0020	ATP-dependent Clp protease regulatory subunit	*clpC*	7	0.71	0.08	0.72^b^	0.03
	slr2076	groEL protein 1	*groL1*	17	1.36^b^	0.04	0.83	0.61
	sll1099	Elongation factor Tu	*tuf*	34	0.86	0.17	0.74^a^	0.10
	sll1712	DNA-binding protein HU	*hup*	6	0.75	0.13	0.50^b^	0.11
	slr1198	Rehydrin	*rehydrin*	14	1.57^a^	0.08	1.55	0.31
	slr1516	Superoxide dismutase [Fe]	*sodB*	31	1.67	0.62	2.46^a^	0.18
Hypotheticals	slr0645	Slr0645 protein	*slr0645*	4	1.23	0.31	2.42^b^	0.32
	slr1619	Slr1619 protein	*slr1619*	2	0.68	0.05	0.39^b^	0.09
	slr2025	Slr2025 protein	*slr2025*	23	1.29^b^	0.06	1.26	0.05

Relative abundance values marked with superscripts: (a) value significant with Bonferroni correction applied. (b) value significant with Bonferroni correction not applied (p values in supplementary materials). Values with no superscript were not deemed significant.

It is instructive to observe that cells acclimated to a lower initial phosphate concentration demonstrate a significantly higher fraction of differentially more abundant proteins from almost all categories (Figure 1), with the exception of proteins associated with the phycobilisomes. These differences suggest a substantial physiological shift within cells in order to maximise P_i_ usage throughout. Indeed, when observing relative abundance levels, the functional categories most broadly affected are metabolism, nutrient acquisition and the proteins of the phycobilisomes (Figure 1, Table 1).

### Nutrient assimilation and regulation

**Figure 2:**
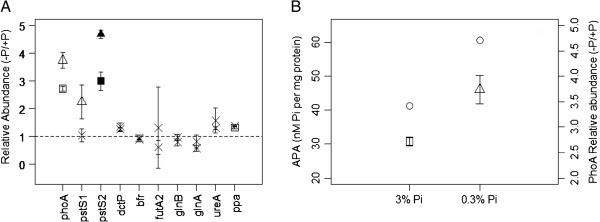
**Nutrient assimilation and regulation. (A)** Relative abundances of proteins involved in nutrient acquisition. Non-significant observations are marked with a cross. Cultures grown in 3% P_i_ are indicated with a square, whilst 0.3% cultures are indicated with a triangle. Empty datum points correspond to significant observations without a Bonferonni correction applied, whereas filled points are observations with the correction. The dotted line represents parity in abundance between control and experimental cultures. (**B**) Levels of measured APase activity (circles) and relative abundances of PhoA within cultures grown in 3% and 0.3% P_i_.

The most commonly reported response to phosphate limitation is an increase in the activity of the periplasmic enzyme alkaline phosphatase [34]. In this study, the alkaline phosphatase activity was observed to increase as the phosphate level decreased, reaching a maximum activity of 60.7 nmol/min · mg in the 0.3% P_i_ culture (Figure 2, Table 2). No alkaline phosphatase activity was detected in the control culture. This finding corresponds well with alkaline phosphatase activity changes observed in other cyanobacteria (*Plectonema boryanum*[35] and *Aphanizomenon ovalisporum*[36]) subjected to long-term phosphate limitation. No acid phosphatase activity was detected in either of the P_i_-limited cultures by our methods.

**Table 2:** Alkaline phosphatase activity within samples

***Phosphate level***	***Phosphate concentration (mg/L)***	***APA (nmoles of Pi released per min per mg of protein)***	***PhoA relative abundance***	***PhoA standard deviation***
**100%**	40	0	na	na
**3%**	1.2	41.3	2.72	0.09
**0.3%**	0.12	60.7	3.74	0.28

The phosphate concentration in the samples was determined using the acid molybdate method [37], and p-nitrophenylphosphate as substrate. Only alkaline phosphatase activity was detected.

Ten proteins involved with nutrient acquisition and regulation (Figure 2A) were quantified. Of these, six of the eight proteins associated with phosphate assimilation and storage were significantly more abundant than in the control (Figure 2A, Table 1). The alkaline phosphatase enzyme, PhoA, and the periplasmic phosphate-binding protein, PstS2, were both significantly more abundant in both 3% and 0.3% Pi cultures, whereas inorganic pyrophosphatase (ppa) was significantly more abundant in 3% Pi culture. We have further confidence with PstS2 abundances, as both data points were significant using the Bonferroni correction. As would be expected, there appears to be no significant observations regarding proteins involved with carbon sources (DctP), iron (bfr) and nitrogen metabolism (GlnA, GlnB, and UreA).

Considering both the relative protein abundance of PhoA in the 100%, 3% and 0.3% P_i_ cultures, and observed APase activity (Figure 2B, Table 2), there is a direct correlation (p < 0.05, where polynomial regressions fitted both datasets with R^2^ values > 0.99, and APase activity is approximately 22.5 x PhoA abundance). To the authors’ knowledge, this is the first observed direct correlation between protein abundances and APase activity within *Synechocystis*.

An interesting observation in relative levels of protein abundances are the significant differences between abundances of proteins in the 3% P_i_ and 0.3% Pi cultures. PhoA, and PstS12 are both significantly more abundant in the 0.3% P_i_ than in 3% P_i_ (Figure 2A), whereas there is no significant difference in abundance of Ppa proteins between the two cultures. This suggests the possibility of a two-tier strategy in cell acclimation: (i) a phased response, whereby protein abundance is putatively associated with/ proportional to internal or external phosphate concentrations, and (ii) a switched response, whereby the cell has a possible biochemical ‘marker’ for activation, for example an as yet undefined external phosphate concentration, and which below this abundance levels of that protein are constitutively maintained within the cell. However, more research is required to investigate this.

It has been previously demonstrated that the two periplasmic phosphate-binding proteins PstS12 have unique enzymatic and regulatory properties [22]. *pstS1* encodes a low-affinity, high velocity P_i_ transporter protein that was expressed to a significantly greater extent than the *pstS2* transcript when cells were grown in P_i_-replete conditions. However, *pstS2*, a high-affinity, low-velocity transporter, was expressed at significantly higher levels under P_i_-stress conditions [22]. In addition, the disruption of *pstS1* caused constitutive expression of the *pho* regulon, indicating a role in external P_i_ sensing [22]. The results here, in part, support those findings. However, our results also infer that elevated expression of *pstS2* is not limited to a stress response, but is also an acclimation response. PstS2 is significantly higher than PstS1 within 3% and 0.3% P_i_ cultures, and the abundance of PstS2 within the 0.3% P_i_ culture is significantly higher than in the 3% P_i_ culture (Figure 2A).

### Light harvesting and photosystems

**Figure 3:**
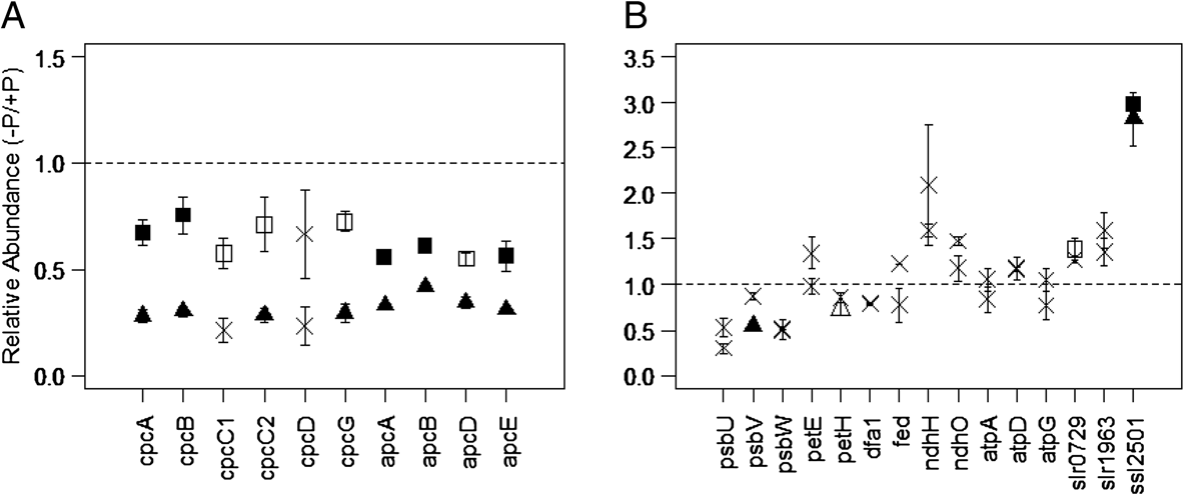
**Protein abundances involved with photosynthesis.** (**A**) Relative abundances of phycobilisome proteins, and (**B**) proteins of the photosystems, PETC, and other thylakoid-related proteins. Non-significant observations are marked with a cross. Cultures grown in 3% P_i_ are indicated with a square, whilst 0.3% cultures are indicated with a triangle. Empty datum points correspond to significant observations without a Bonferonni correction applied, whereas filled points are observations with the correction. The dotted line represents parity in abundance between control and experimental cultures.

Light harvesting within *Synechocystis* is accomplished through large multi-protein complexes, called phycobilisomes, consisting of phycobiliproteins (PBPs). Phycobilisomes are composed of a core complex surrounded by a tiered array of pigmented allophycocyanins, phycocyanins and non-pigmented linker peptides that harness and siphon the light energy to PSII [37]. These structures are significantly affected by prolonged exposure to low P_i_ levels in the medium (Figure 3A, Table 1). Within cyanobacteria, phycobilisome loss varies between species, and specifically under different nutrient deprived conditions. For example, a significant reduction of cyanobacterial phycobilisome content, known as chlorosis, is well known during nitrogen deprivation [38,39], and yet a recent proteomic study of *Synechocystis* during CO_2_ limitation showed a minimal decrease in phycobiliprotein abundance [40]. However, that result is in contrast to previous transcriptome experiments [41,42]. Nevertheless, a study of *Synechococcus* levels of bleaching varied significantly between growth in nitrogen, sulphur and phosphorus deficient media, possibly reflecting differentially regulated subprocesses [43]. Interestingly, Wegener *et al*., [44] demonstrated that, after 6 days of P_i_ depletion, phycobilisomes and pigment biosynthesis were unaffected, yet their cells demonstrated significant chlorosis. This is unexpected and in contrast to our results, in which significant reduction of phycobilisome structures corresponded with chlorosis. It is difficult to immediately explain this disparity; however, the difference in timescales between both experiments may hold a clue.

It is interesting to note the significantly linear relationship within the phycocyanin group and allophycocyanin group of proteins. A statistical analysis of the phycobiliprotein abundances between the 3% and 0.3% P_i_ cultures revealed that there is no significant difference between the phycocyanins (CpcA-G, Figure 3A) and the allophycocyanins (ApcA-E, Figure 3A) at the 3% P_i_ level (p = 0.09, df = 32). However, there is a significant difference at the 0.3% P_i_ level (p = 0.006, df = 32). This difference indicates that the light harvesting antennae of 0.3% P_i_ culture cultures have significantly less of the phycocyanins in proportion to their allophycocyanin content than do the 3% P_i_ culture cultures. This potentially suggests independent regulatory systems between the different PBP groups, or differential regulation of group specific proteases, whose activity is putatively dependent upon external P_i_ concentrations. This is a reasonable hypothesis, as phycocyanins are attached externally to allophycocyanins through linker peptides, and light energy is funnelled from phycocyanins through allophycocyanins to PSII [37]. This regulation may be due to the organism attempting to reduce photosynthetically induced oxidative damage.

Four proteins within photosystem II (PSII) and the photosynthetic electron transport chain (PETC) are differentially less abundant (Figure 3B). Both PsbU and PsbW appear to be less abundant than in the control culture under both conditions, whereas PsbV is significantly less abundant only in the 0.3% P_i_ culture. The extrinsic protein PsbV, alongside PsbU, is a critical protein in the oxygen evolving complex of cyanobacterial PSII complexes [45,46]. Indeed, PsbU appears to promote stability within the water-splitting PSII structure [47]. PsbW (characterized recently, and more commonly referred to as Psb28 within *Synechocystis*[48]) appears to be closely associated with PSII complexes, and more specifically to the biogenesis of the chlorophyll-binding CP47 protein [49]. As a consequence, it appears that when cells acclimate to low levels of ambient P_i_, light harvesting and structural integrity of the PSII assembly is compromised.

Ferridoxin NAPDH reductase (PetH, Figure 3B) is commonly associated with PSI and the terminal end of the PETC, nominally with regards to the production of reducing energy. However, in *Synechocystis* it has also been demonstrated to be closely associated with the phycobilisomes [50]. Within our study, the relative abundance of PetH is significantly less within the 0.3% P_i_ culture, but not the 3% P_i_. This then, coupled with the PsbV observation, implies a strategic down regulation of PETC and PSII activity as ambient P_i_ concentrations decrease. However, no other proteins associated with PSI were identified, and the suggestion that PSI function was decreased requires further validation in the future.

It is clear that ambient P_i_ concentrations have a significant effect upon the photosynthetic machinery within *Synechocystis*. However, it is also clear that this did not mean a complete loss of function, as occurs during prolonged nitrogen deprivation [38], and represents a proportional loss of function, dependent upon P_i_ concentrations. Our results present evidence of a response comparable to *Synechococcus* sp. PCC 7942 [43].

### Central metabolism

**Figure 4:**
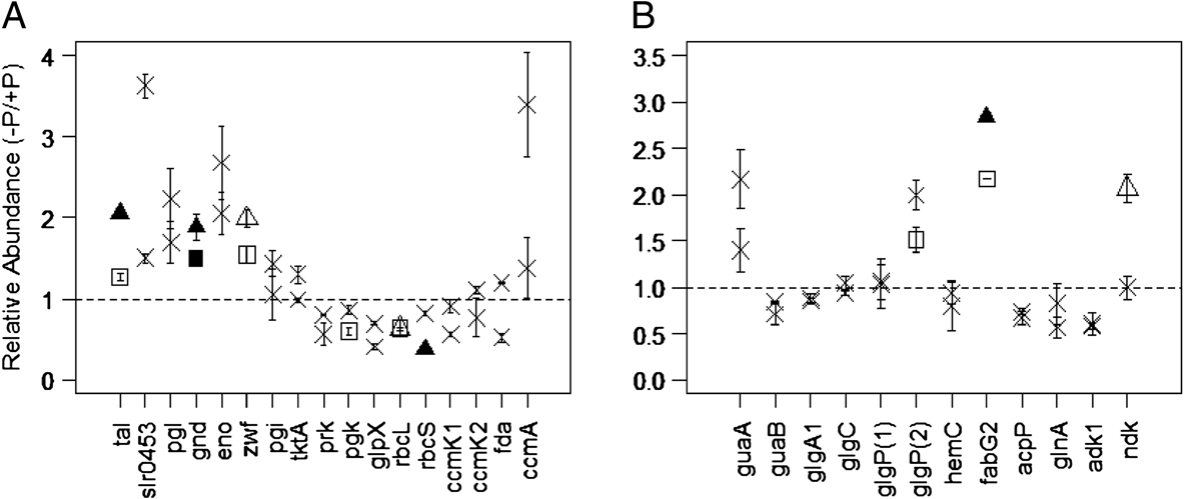
**Relative abundances of proteins involved in (A) central metabolic processes, and (B) biosynthesis.** Non-significant observations are marked with a cross. Cultures grown in 3% P_i_ are indicated with a square, whilst 0.3% cultures are indicated with a triangle. Empty datum points correspond to significant observations without a Bonferonni correction applied, whereas filled points are observations with the correction. The dotted line represents parity in abundance between control and experimental cultures.

The central metabolism of *Synechocystis* was affected by prolonged exposure and acclimation to reduced P_i_ concentrations. Ten proteins were identified as being significantly differentially abundant in both 3% P_i_ and 0.3% P_i_ compared to the control cultures (Figure 4A, Table 1). Three proteins that were more abundant than the control (in both% P_i_ and 0.3% P_i_ cultures) were transaldolase (Tal), 6-phosphogluconate dehydrogenase (decarboxylating) (Gnd) and glucose-6-phosphate 1-dehydrogenase (Zwf) (Figure 4A). These proteins function within the 6^th^, 3^rd^ and 1^st^ steps in the pentose phosphate pathway (PPP) respectively. Allied to this, 6-phosphogluconolactonase (Pgl), the 2^nd^ step in PPP, demonstrated a strong indication that it was more abundant in both the 3% and the 0.3% cultures (Figure 4A), but this is not clear. This is an important observation, as the PPP acts to generate reducing energy (in the form of NADPH), and also generates metabolic precursors for nucleotide/nucleic acid and amino acid biosynthesis [51].

In contrast, three glycolysis and carbon fixation proteins, phosphoglycerate kinase (Pgk) and the two subunits of rubisco (RbcL and RbcS) were significantly less abundant in 3% P_i_ and 0.3% P_i_ vs the 100% P_i_ controls (Figure 4A, Table 1). These proteins are the central enzymes within the first two phases of the Calvin-Benson cycle (being the fixation of CO_2_, the reduction of 3-phosphoglycerate, and the regeneration of ribulose 1,5-bisphosphate). This cycle is critical in all photosynthetic organisms, and is fundamentally limiting to growth and reproduction. This result is unsurprising, given the significant reduction in photosynthetic light harvesting earlier observed in both 3% and 0.3% P_i_ cultures. It is also interesting to note that the enzyme Transketolase (TktA), which functions in both the Calvin-Benson cycle and PPP was not differentially abundant.

Interestingly, at the transcript level, *gnd*, *tal* and *zwf* were all strongly induced under nitrogen deprivation, as well as one of the two glycogen phosphorylases *glpP* (*sll1367*). The Calvin-Benson cycle genes *rbcL* and *rbcS* were also down regulated [52]. Our results demonstrate that the other glycogen phosphorylase (sll1356), rather than sll1367 is more abundant (see below), however the similarity in results is noteworthy. Osani and co-workers [52] concluded that nitrogen deprivation induces sugar catabolism, and that the PPP is the primary method of reducing power production. Our results confirm that P_i_ deprivation also leads to a similar biochemical response, and thus indicate that the central metabolic response of the cell is similar for both N and P_i_ deprivation. However this response has a significantly different level of severity depending upon N or P_i_ deprivation [43].

When considering energy metabolism (defined in this instance as the synthesis and degradation of the glycolytic/PPP precursor glucose 6-phosphate), Figure 4B shows a significant difference between the relative abundance of two glycogen phosphorylation proteins (GlgP(1) & GlgP(2)). Both proteins are assumed to function to degrade glycogen, thereby generating D-glucose-1-phosphate in the preparatory, rate-limiting step before entry into glycolysis. However, the differential relative abundance between both proteins suggests differential functional roles within the cell. The enhancement of central PPP protein abundances highlighted earlier suggests that glycogen is being processed by GlgP(2) to support the production of NADPH and pentose sugars. As mentioned earlier, GlgP(1) rather than GlgP(2) was found to be differentially upregulated at the transcript level when subjected to N deprivation [52], which then suggests differing regulatory controls for each enzyme and condition. Also, Wegener and associates observed that sll1367 was more abundant than sll1356 under P deplete conditions [44], and so we must conclude that over short P starvation periods, sll1367 is preferred, and over longer, chronic, periods sll1356 predominates.

### Transcription, translation, stress and hypothetical proteins

Six proteins out of 24 relating to transcription, translation and stress were significantly differentially abundant within both reduced P_i_ cultures. An interesting group of proteins are the ATP-dependant Clp proteases, ClpC, ClpP1, ClpP2 and ClpB. These proteins are vital for growth and stress acclimation, and act to degrade denatured polypeptides and maintain cellular homeostasis [53]. Within *Escherichia coli* there is evidence that these proteins act in a multi-subunit complex, with the exclusion of ClpB (which acts independently) [54]. However, little is known about cyanobacterial Clp proteases, except that cyanobacterial ClpC is 90% similar to the ClpC in plant chloroplasts, is transcriptionally constitutively expressed at a level which appears to be unaffected by stress conditions, and that loss-of-function manipulation is generally fatal [55]. This last point emphasises the protein’s role as an essential chaperone.

**Figure 5:**
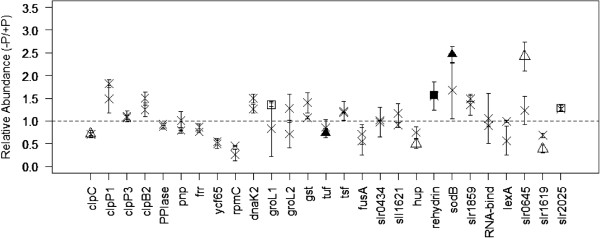
**Relative abundances of proteins involved with transcription, translation, stress and uncharacterised hypothetical proteins.** Non-significant observations are marked with a cross. Cultures grown in 3% P_i_ are indicated with a square, whilst 0.3% cultures are indicated with a triangle. Empty datum points correspond to significant observations without a Bonferonni correction applied, whereas filled points are observations with the correction. The dotted line represents parity in abundance between control and experimental cultures.

As such, it is interesting that the regulatory subunit, ClpC, was significantly lower in both reduced P_i_ cultures, and yet one of the two identified isomeric forms of ClpP, ClpP1, demonstrated no significant differential abundance (Figure 5). While this seems counterintuitive, later studies showed that ClpC does not interact with ClpP1 or ClpP2, but rather with ClpP3 and ClpP4. As such, two distinct and separate Clp protease complexes function within *Synechococcus elongates* PCC 7942 [56], which indicates that each complex functions to degrade differing substrates. At this time, the substrate for ClpC is unknown. Considering the other chaperone proteins identified, only GroL1 was differentially abundant (Figure 5). This has been observed within *Prochlorococcus marinus* MED4 during high-light stress [57], but was not observed within MED4 during acclimation to low P_i_[18]. This abundance difference between species and environmental conditions is puzzling, and likely reflects other, unknown, contributory factors.

Translation appears to be affected as the elongation factor, EF-Tu (Tuf, Figure 5), which facilitates the entry of aminoacyl tRNA into the free site of the ribosome, was significantly lower in abundance within the 0.3% culture. EF-Tu is critical in successful translation, and acts as a powerful indicator that translation is lower within cells acclimated to long term P_i_ deprivation. It is instructive to note that the other three elongation factors identified in our study (Gst, Tsf, and FusA) were unaffected, which may indicate a regulatory role for EF-Tu.

Two known stress response proteins were more abundant in low P_i_: rehydrin and SodB (Figure 5). The functional role of rehydrin, a protein associated with desiccation stress in mosses [58], is unknown, and this represents the first recording of this protein being intrinsic to P_i_ acclimation. In contrast, the function of the iron superoxide dismutase protein, SodB, is well characterised [59], and is primarily responsible for the cell’s response to oxidative stress.

One biosynthetic protein was significantly differentially abundant within the P_i_-deplete cultures than the control, 3-oxoacyl-[acyl-carrier protein] reductase (FabG2, Figure 4B, Table 1), which participates in fatty acid biosynthesis. This is interesting as fatty acids play two main functions within the cell: (i) they are precursors of membrane lipids, and (ii) they accumulate for energy storage. However given their differential abundance, it would be expected that another critical protein required in this process, the acyl-carrier protein (acp) demonstrates no differential abundance in either the 3% P_i_, or the 0.3% P_i_ culture. The reason for this is unclear, however the abundance of FabG2 is a strong indication that the cell over produced fatty acids when acclimating to low P_i_ environments. Indeed, fatty acids have been speculated to have a role in stress tolerance within cyanobacteria, notably to desiccation, salt and temperature stress (see Singh *et al*. [60] for review). As well as energy storage, the production of lipids may help to stabilize nutrient acquisition machinery within the external membrane [60], and replace lipids damaged by reactive oxygen species, which have concurrent effects upon membrane fluidity and other cellular metabolic functions [61]. The role of fatty acids in desiccation stress is intriguing, specifically due to the increased abundance of the protein rehydrin within the 3% culture (Figure 5, Table 1).

Twenty-one hypothetical proteins were identified within this study (Additional file 2: Table S1). Although at the time of writing, the functions of particular hypothetical proteins are unknown, it is an important observation that three proteins are differentially abundant within the cultures within this study. They are slr0645, slr1619 and slr2025 (Figure 5, Table 1). Of the three, slr2025 was recently observed within a proteome-wide survey of *Synechocystis* under CO_2_ limitation. However, it was not considered differentially abundant [42], and so appears to be unimportant to acclimation to that environment. In our study, it was more abundant within the 3% P_i_ culture.

## Conclusions

Since *Synechocystis* typically inhabit rivers and lakes where the ambient P_i_ concentrations range from nanomolar to micromolar ranges [62], our control cultures were acclimated to “luxury” P_i_ concentrations, and hence the 3% and the 0.3% P_i_ cultures, once re-acclimated, are more indicative of natural conditions. This study then emphasises the physiological mechanisms important for normal cellular reproduction and growth. The observed proteome acclimation response to environmental phosphate concentrations involved shifts in cellular abundances of P_i_ acquisition and storage mechanisms, coinciding with changes in its photosynthetic machinery and a shift in central metabolism towards the production of reducing energy. While the coordination and control of this response was not uncovered, we and others have previously speculated that it is likely to be a complex interaction of internal regulatory systems initiated through the *phoBR* homologue of *Synechocystis* – the SphS-SphR two component system [10], and the activity of PstS1 [22].

We have shown that the observed abundance response of alkaline phosphatase is proportional to measured APase activity, and interestingly that there appears to be a coordinated and specific reduction of individual phycobiliprotein groups during acclimation. As has been previously reported for higher plants, these results provide some evidence that intracellular P_i_ concentrations control photosynthetic activity in *Synechocystis* through the activation or deactivation of RuBisCO at different concentrations [63]. The similarity of the *Synechocystis* response to P_i_ deprivation appears to be remarkably similar to prolonged nitrogen deprivation, although the magnitude of the response is greater in low nitrogen than in low P_i_. In both cases, the photosynthetic machinery was reduced significantly, and central metabolic processes shift to produce not just reducing energy but nucleotide sugars. We hypothesise that this cellular shift is designed to function in two ways: 1) that an increase in reducing energy provides the ‘power’ for the cell to stabilize and maintain the cell membranes, and 2) nucleotide sugars can be readily used as precursors for both ATP and GTP, as well as enabling the cell to quickly respond to changes in the environment through rapid production of RNA. The cell is therefore able to respond, grow and replicate rapidly.

## Materials and methods

### Growth conditions

*Synechocystis* was principally grown in BG-11 medium [64] in 1 L conical flasks with a 0.5-L working volume shaken at 100 rpm. The P_i_ replete BG-11 medium contained 40 mg/L of K_2_HPO_4_. For phosphate depletion experiments, the concentration of K_2_HPO_4_ within BG-11 was prepared as 3% w/v (1.2 mg/L) and 0.3% w/v of the control P_i_ replete BG-11 medium. The experimental design utilised biological duplicates of *Synechocystis* PCC 6803 cultured in each condition: (A) 100% P_i_ (control), (B) 3% P_i_, and (C) 0.3% P_i_. Inocula for each culture were prepared by initial centrifugation of stock cultures followed by washing cells twice with sterilized deionised water and once with the respective P_i_ reduced medium, prior to resuspension into the respective medium and experimental inception. Cultures were incubated in a Sanyo MLR-350H environmental chamber at 25°C on a 12-h light–dark cycle for 60 days with an initial optical density of 0.2. Irradiance was provided by cool-white fluorescent lamps at an incident intensity of 70 μE m^-2^ s^-1^ as measured by a three-dimensional QSL-2100 Light Sensor (Biospherical Instrument Inc, San Diego, CA). The growth rate was monitored and recorded using an Ultraspec 2100-Pro spectrophotometer (Biochrom, Cambridge, UK) at an OD of 730 nm.

### Protein extraction and quantitation

Cells were harvested at day 60 by centrifugation at 5,000 × g for 5 min at 4°C. The cell pellets were re-suspended in 500 mM triethylammonium bicarbonate buffer (TEAB) at pH 8.5 and proteins were extracted through mechanical cracking with liquid nitrogen [65]. Soluble proteins were recovered from the supernatant by centrifugation at 21,000 × g for 30 min at 4°C. The total soluble protein concentrate was then measured using the RC DC Protein Quantification Assay (Bio-Rad, Hertfordshire, UK) according to the manufacturer’s protocol.

### Acid/alkaline phosphatase assays

A subsample of cells broken by mechanical grinding with liquid nitrogen as described above was subsequently re-suspended in Tris–HCl buffer (pH 8.0) containing 5 mM MgCl_2_. Alkaline phosphatase assays were performed in duplicate as described by O’Loughlin *et al*. [66] at 30°C. The assay solution contained 50 mM Tris–HCl (pH 8.0), 20 mM MgCl_2_, and 5 mM p-nitrophenyl phosphate with 0.01-0.1 mg cell-extract protein in a final reaction volume of 1 mL. For acid phosphatase assays, the Tris–HCl buffer was replaced with 50 mM succinate-NaOH buffer (pH 5.0).

Due to the presence of large quantities of chlorophylls in the cell-extract, detection of p-nitrophenol production by means of the widely used absorbance measurement at 412 nm was not possible. Reactions were therefore quenched with 0.2 vol of 3 M trichloroacetic acid solution following addition of cell extract protein and a duplicate reaction tube quenched 10 min later. The resultant reaction supernatants were cooled on ice for 10 min prior to centrifugation at 20,000 × g for 5 min to remove precipitated protein, following which the phosphate concentration in the samples was determined using the acid molybdate method of Fiske and SubbaRow [67]. ‘No substrate’ and ‘no cell-extract’ controls were included and activity was expressed as nmol of P_i_ released per min per mg of cell-extract protein, taking the control reactions into account.

### SCX prefractionation and iTRAQ

Approximately 100 μg of protein from each sample was placed in PCR clean LoBind microcentrifuge tubes (Eppendorf, Cambridgeshire, UK), then reduced, alkylated and digested as previously reported [18]. The resultant peptide digests were then labelled using 6 labels from an 8-plex iTRAQ chemical reagents kit (AB Sciex UK Limited, UK), with phosphate replete control samples (nominated as 100% P_i_) tagged with the 113 and 114 labels, cells grown in 3% P_i_ tagged with 115 and 116, and the 0.3% phosphate-grown cells tagged with the 117 and 118 labels. Primary peptide fractionation was carried out through strong cation exchange (SCX) on a BioLC HPLC unit (Dionex, Surrey, UK) with a Poly SULFOETHYL A™ column (PolyLC, Columbia, MD, USA). The column used 5 μm particle size, and had dimensions of 200 mm length with an internal diameter of 2.1 mm and a 200 Å pore size. The buffers used were Buffer A (10 mM KH_2_PO_4_ with 20% HPLC grade acetonitrile (ACN) at pH 2.5) and Buffer B (10 mM KH_2_PO_4_ with 20% HPLC grade acetonitrile (ACN) and 500 mM KCl at pH3) with a gradient of 0% B for 5 min, 0-40% B for 30 min, 40-100% B for 10 min, 100% B for 5 min, and 0% B for 5 min. Before fractionation, the digests were dried in a vacuum centrifuge and resuspended in 200 μL Buffer A. In order to remove salts and unbound iTRAQ reagents, samples were injected onto the column, and manual acquisition was initiated. This allowed Buffer A to flow through the column, leaving bound peptides attached and purging all non-bound material. Two injections were completed before initiation of the gradient. The flow rate was maintained at 200 μL min^-1^. A UV detector (UVD170U) was used to monitor the fractionation process, and the entire operation was controlled and through Chromeleon software (Dionex/LC packings, the Netherlands). Fractions were collected each minute for the duration of the run with a Foxy Jr. Fraction Collector (Dionex). Fractions were selected for subsequent MS/MS analysis according to SCX chromatogram intensity.

Mass spectrometric analysis was performed on a QStar XL Hybrid ESI Quadrupole time-of-flight tandem mass spectrometer, ESI-qQ-TOF MS/MS (Applied Biosystems, Framingham, MA; MDS-Sciex, Concord, Ontario, Canada), coupled with an Ultimate 3000 online capillary liquid chromatography (LC) system (Applied Biosystems, Framingham, MA), with a PepMap C-18 RP column (LC Packings) set to a constant flow rate of 0.3 μL min^-1^. Selected fractions were dried and resuspended in 10 μL of Buffer A prior to analysis. To prevent salts, and other contaminants entering the mass spectrometer, a C-18 column clean up (The Nest Group, Inc. Southborough, MA) of each fraction was performed. Cleaned samples were then injected into the online nanoflow LC-MS/MS unit where the buffers were Buffer A (3% (v/v) ACN with 0.1% (v/v) formic acid (FA)), and Buffer B (97% (v/v) ACN with 0.1% (v/v) FA). The gradient applied for the second dimension of chromatography was 3% Buffer B for 3 min, 3 – 40% Buffer B for 70 min, 90% Buffer B for 5 min, and 3% Buffer B for 7 min. The data acquisition protocol in the mass spectrometer was set to positive ion mode, and the addition of formic acid to the buffers was used in order to promote protonation. Peptides were selected by the mass spectrometer for further tandem MS/MS due to observed charge states of +2 and +3.

### Data analysis

Mass spectrometry data analysis, peak list conversion, peptide identification and quantitation was carried out using the combination of Analyst QS ver. 1.1 (ABSciex, Foster City, CA) and Phenyx 2.6 (Geneva Bioinformatics, Geneva, Switzerland) software against the UniProt *Synechocystis* (accessed May 2010). The databases used for searching were Synechocystis PCC6803 (including plasmids), and the NCBI non-redundant database (http://www.ncbi.nlm.nih.gov/, Accessed 01/07/10). Searches of the *Synechocystis* database were conducted using a target-decoy approach (reversed sequence decoy). All searches were performed with the parameter settings: 0.1 Da MS scan tolerance, 0.15 Da MS/MS scan tolerance, strict trypsin cleavage with one allowable missed cleavage and a minimum of 20% of combined y, y^2+^, b, b^2+^ ions. Acceptance threshold for peptide identification were set to p-value ≤ 1 × 10^-4^ with a peptide score ≥ 5. Protein identification was then set to include only matches with at least a score of 7 (AC score ≥ 7).

Statistical inference was obtained using a *t*-test comparison between the reporter ions’ intensities, as previously detailed in Mukherjee *et al*. [68] and Pham *et al*. [69]. Within this study, we include observations made with and without a Bonferonni correction.

A principal component analysis (PCA) and a hierarchical clustering were performed on the proteomic data using *Mathematica* (Wolfram Research) so as to provide an appreciation of biological/stress variation (see Additional file 1: Figures S3 and Additional file 1: Figure S4). These two analyses were carried out on proteins quantified with three or more MS/MS scans. For the PCA, the first two principal components were used; the clustering analysis used Ward’s linkage method.

## Competing interests

The authors declare that they have no competing interests.

## Authors’ contribution

CAB, GM, KFR & PCW conceived and designed the experiment, MAF, CSG & SYO carried out the proteomic experiments and data analysis, JN carried out statistical analysis of the data, NT & GM conducted the enzyme assays. All authors contributed to the writing of the manuscript. All authors read and approved the final manuscript.

## Supplementary Material

Additional file 1: Figure S1 Growth curves for *Synechocystis* PCC6803 grown in P_i_ replete medium (green circles), 3% P_i_ medium (red triangles), and 0.3% P_i_ medium (purple diamonds). All cultures were in early stationary phase upon harvesting. **Figure S2.** Representation of the broad functional categories associated with *Synechocystis* PCC6803 proteins identified in this study. **Figure S3.** Hierarchical clustering of the proteomic data (log-ratios). We only used proteins for which three or more MS/MS scans were identified; Ward’s linkage method was used. This shows good reproducibility of our samples. **Figure S4.** Principal component analysis of the proteomic data (log-ratios). The first two principal components are shown here (PC1 abscissa and PC2 ordinate). We only used proteins for which three or more MS/MS scans were identified. Click here for file

Additional file 2: Table S1 Protein list. Click here for file
