# Supplementary material for: The quantitative proteomic response of Synechocystis sp. PCC6803 to phosphate acclimation
Source: Aquat Biosyst. 2013 Feb 26;9:5. doi: 10.1186/2046-9063-9-5 (PMC3600050; doi:10.1186/2046-9063-9-5)
Supplement: Additional file 1: Figure S1 — Growth curves for Synechocystis PCC6803 grown in Pi replete medium (green circles), 3% Pi medium (red triangles), and 0.3% Pi medium (purple diamonds). All cultures were in early stationary phase upon harvesting. Figure S2. Representation of the broad functional categories associated with Synechocystis PCC6803 proteins identified in this study. Figure S3. Hierarchical clustering of the proteomic data (log-ratios). We only used proteins for which three or more MS/MS scans were identified; Ward’s linkage method was used. This shows good reproducibility of our samples. Figure S4. Principal component analysis of the proteomic data (log-ratios). The first two principal components are shown here (PC1 abscissa and PC2 ordinate). We only used proteins for which three or more MS/MS scans were identified. [file 2046-9063-9-5-S1.doc]

**The quantitative proteomic response of *Synechocystis* sp. PCC6803 to phosphate acclimation, Fuszard et al.**

**Supplemental Materials**


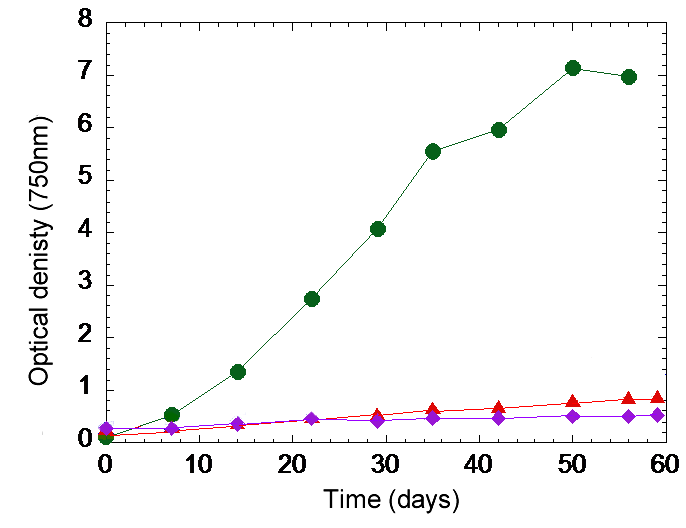


**Figure S1** Growth curves for *Synechocystis* PCC6803 grown in P_i_ replete medium (green circles), 3% P_i_ medium (red triangles), and 0.3% P_i_ medium (purple diamonds). All cultures were in early stationary phase upon harvesting.


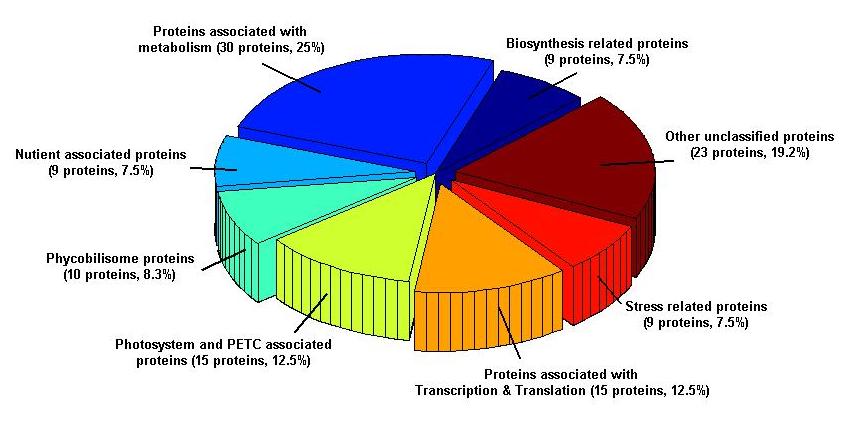


**Figure S2** Representation of the broad functional categories associated with *Synechocystis* PCC6803 proteins identified in this study.


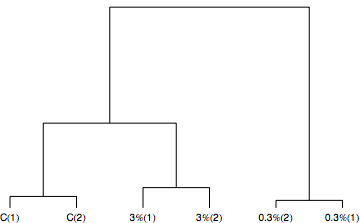


**Figure S3.** Hierarchical clustering of the proteomic data (log-ratios). We only used proteins for which three or more MS/MS scans were identified; Ward’s linkage method was used. This shows good reproducibility of our samples.


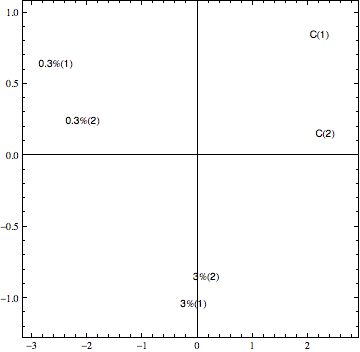


**Figure S4.** Principal component analysis of the proteomic data (log-ratios). The first two principal components are shown here (PC1 abscissa and PC2 ordinate). We only used proteins for which three or more MS/MS scans were identified.
